# E2F1 promotes cell migration in hepatocellular carcinoma via FNDC3B

**DOI:** 10.1002/2211-5463.13783

**Published:** 2024-02-25

**Authors:** Kate Hua, Chen‐Tang Wu, Chin‐Hui Lin, Chian‐Feng Chen

**Affiliations:** ^1^ Cancer Progression Research Center National Yang Ming Chiao Tung University Taipei Taiwan

**Keywords:** ChIP, ddPCR, E2F1, FNDC3B, hepatocellular carcinoma, transcription factor

## Abstract

FNDC3B (fibronectin type III domain containing 3B) is highly expressed in hepatocellular carcinoma (HCC) and other cancer types, and fusion genes involving FNDC3B have been identified in HCC and leukemia. Growing evidence suggests the significance of FNDC3B in tumorigenesis, particularly in cell migration and tumor metastasis. However, its regulatory mechanisms remain elusive. In this study, we employed bioinformatic, gene regulation, and protein‐DNA interaction screening to investigate the transcription factors (TFs) involved in regulating FNDC3B. Initially, 338 candidate TFs were selected based on previous chromatin immunoprecipitation (ChIP)‐seq experiments available in public domain databases. Through TF knockdown screening and ChIP coupled with Droplet Digital PCR assays, we identified that E2F1 (E2F transcription factor 1) is crucial for the activation of FNDC3B. Overexpression or knockdown of E2F1 significantly impacts the expression of FNDC3B. In conclusion, our study elucidated the mechanistic link between FNDC3B and E2F1. These findings contribute to a better understanding of FNDC3B in tumorigenesis and provide insights into potential therapeutic targets for cancer treatment.

## Graphical abstract

FNDC3B is highly expressed and involved in cell migration. To explore its regulation mechanism, we utilized bioinformatic, regulation, and DNA–protein interaction screening to investigate its upstream regulator from 338 candidates. Our results revealed that E2F1 binds to the promoter and modulates the expression and function of FNDC3B. These findings enhance our understanding of the regulatory mechanisms governing FNDC3B expression.

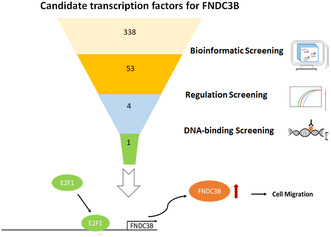

**Abbreviations**
ChIP: chromatin immunoprecipitation
ddPCR: droplet digital PCR
E2F1: E2F transcription factor 1
FNDC3B: fibronectin type III domain containing 3B
HCC: hepatocellular carcinoma
qPCR: quantitative PCR
TF: transcription factors

FNDC3B (fibronectin type III domain containing 3B) exhibits high expression level in hepatocellular carcinoma (HCC) and other cancers types [1, 2, 3], and its gene locus, 3q26, is frequently amplified in various cancer genome [4, 5]. Fusion genes involving FNDC3B have been identified in HCC and leukemia [6, 7, 8]. Growing evidence suggests the significance of FNDC3B in tumorigenesis, particularly in cell migration and tumor metastasis. *In vitro* studies have demonstrated that FNDC3B induces the epithelial‐to‐mesenchymal transition, thereby enhancing cell migration [9, 10, 11]. *In vivo* metastasis experiments have revealed that the knockdown of FNDC3B reduces tumor nodule formation [9]. Clinical analysis of cancer patients' disease‐free survival has indicated that overexpression of FNDC3B is associated with recurrence and metastasis in various cancer types [9, 10, 11]. Additionally, several tumor suppressor miRNAs have been shown to inhibit cell migration by targeting FNDC3B [12, 13, 14]. Despite the accumulating evidence highlighting the importance of FNDC3B in tumorigenesis, its regulation mechanisms remain largely unknown.

Transcription factor (TF) is a kind of DNA‐binding protein responsible for regulating transcription [15, 16]. Their binding to DNA can directly activate or repress gene promoters and influence the expression of specific gene [17, 18]. Various experimental assays have been developed to determine the binding sites of TFs [19]. Chromatin immunoprecipitation (ChIP) is one of the most powerful techniques for evaluating the interactions of proteins with specific regions of genomic DNA [20, 21]. Coupled with next‐generation sequencing technologies, ChIP‐seq permits the genome‐wide identification of TF binding sites [22]. After mapping ChIP‐seq reads to the genome, peak calling algorithms are employed to predict the genomic binding locations for the factor [23]. In recent years, metadata of ChIP‐seq produced from different research groups have been integrated into several public domain databases, such as ECODE and GTPD [24, 25]. These databases provide a valuable tool for exploring potential TFs for specific gene of interest.

In this study, we collected TF information for FNDC3B from public domain databases. By combining gene regulation and DNA‐binding assay, we systematically identified E2F1 as a transcription activator for FNDC3B. Our findings demonstrate that E2F1 regulates the expression and function of FNDC3B.

## Materials and methods

### Cell culture and cell lines

HCC cell lines, HepG2, HuH7, and SKhep1 were generous gifts from Dr. Yuh‐Shan Jou (Academia Sinica, Taipei, Taiwan). They were cultured in Dulbecco's modified Eagle's medium supplemented with 10% fetal bovine serum, 1% nonessential amino acids, and 1% penicillin/streptomycin (Thermo Fisher Scientific, Waltham, MA, USA).

### Exploring potential TFs for FNDC3B

ENCODE ChIP‐seq data from 338 TFs are combined into clusters to produce a summary display showing occupancy regions for each factor [26]. All the binding information is available on the website of UCSC Genome Browser website (http://genome.ucsc.edu). Candidate TFs were selected for their occupancy regions within 5 kb upstream of the transcription start site.

### RNA interference (RNAi) knockdown assay for TF screening

Short hairpin RNAs (shRNAs) targeting TFs in the RNAi Consortium shRNA library were ordered from the National RNAi Core Facility (Academia Sinica). We selected two effective shRNAs to knock down the expression of E2F1 (TRCN0000332897 and TRCN0000332898), MAFF (TRCN0000016450 and TRCN0000016451), and MAX (TRCN0000231551 and TRCN0000231550) by 293 T‐produced lentivirus infection. The targeted cells were then incubated with lentiviruses for 24 h with 8 μg·mL^−1^ of polybrene (Sigma‐Aldrich, St. Louis, MO, USA). Then total RNA from TF knockdown cells was extracted via TRIzol reagent (Thermo Fisher Scientific) according to the manufacturer's protocol. The mRNA expression of FNDC3B was obtained by normalizing the FNDC3B quantification cycle (*C*_t_) values for FNDC3B to GAPDH Ct values. The primers and Taqman probes (FNDC3B: Hs00981553_m1, GAPDH: Hs02786624_g1) for qPCR analysis were obtained from Thermo Fisher (Thermo Fisher Scientific).

### Chromatin immunoprecipitation ‐ droplet digital PCR (ChIP‐ddPCR) assays

Chromatin immunoprecipitation (ChIP) evaluates TF‐DNA interactions and is critical for advancing gene expression regulation and epigenetic modification studies. The genomic DNA is cross‐linked with DNA‐binding proteins and sonication down to desired fragment size. The antibodies against E2F1 (#3742, Cell Signaling Technology, Danvers, MA, USA), MAX (#85570, Cell Signaling Technology), and IgG (ab172730, Abcam, Cambridge, UK) were coupled to magnetic beads (Dynabeads, Thermo Fisher Scientific) to capture protein‐DNA complexes. Then, the DNA fragments were analyzed by QX200 ddPCR system (Bio‐Rad, Hercules, CA, USA). The sample, primers, supermix, and mineral oil were loaded into a droplet generator to form thousands of droplets. According to the manufacturer's protocol, PCR was performed with the primer pairs designed for E1 (Forward: CTG CGA GTC TGC GAT TGT and Reverse: GAT TCC GCC AAT GTC AAT GAT G), E2 (Forward: CTA TTG TGG TCC TTG CAC TG and Reverse: CTC ACC CAG CCT TAT TTC AC), and control (Forward: GTC AGA TAT GGC TCA GGA CAT T and Reverse: GGG CAA CAA GAG CAA ACT TC). Droplets were then aspirated and read by the Droplet Reader. Data were analyzed by quantasoft analysis software (Bio‐Rad).

### Luciferase reporter assay

The FNDC3B promoter sequence (−971 to +4) was cloned into pGL4 plasmid (Promega, Madison, WI, USA). The derivative mutates for predicted E2F1 binding sites vector using the point mutation kit (Agilent, Santa Clara, CA, USA). The pGL4 empty plasmid was used as a negative control. After incubation for 24 h, cells which transfected with reporter plasmid were collected and analyzed for luciferase activity with Dual‐Luciferase Reporter Assay System (Promega).

### Cell migration assays

The migration assay was performed in 24‐well transwell units with an 8 mm pore size polycarbonate membrane (BD Biosciences, San Jose, CA, USA). Cells resuspended in a serum‐free medium were seeded into the upper chamber of the insert and then placed into the bottom chamber containing 10% fetal bovine serum as a chemoattractant. Cells were allowed to migrate for 24 h, followed by methanol fixation and Giemsa staining (MERCK, Darmstadt, Germany). Cells that did not migrate to the apical side of the membrane were removed with a cotton swab. Migrated cells were photographed with a phase contrast microscope. All experiments were performed in triplicates. Independent Student's *t*‐tests were used to compare the continuous variables between the two groups.

### Statistical analysis

Statistical analysis was performed using graphpad prism (v.8.0.2) (GraphPad Software, Boston, MA, USA). Comparison of the expression of E2F1 between HCC and normal groups was performed using Wilcoxon rank sum tests, and adjacent groups with Wilcoxon signed‐rank tests.

## Results

### Bioinformatic screening for candidate TFs

**Fig. 1:**
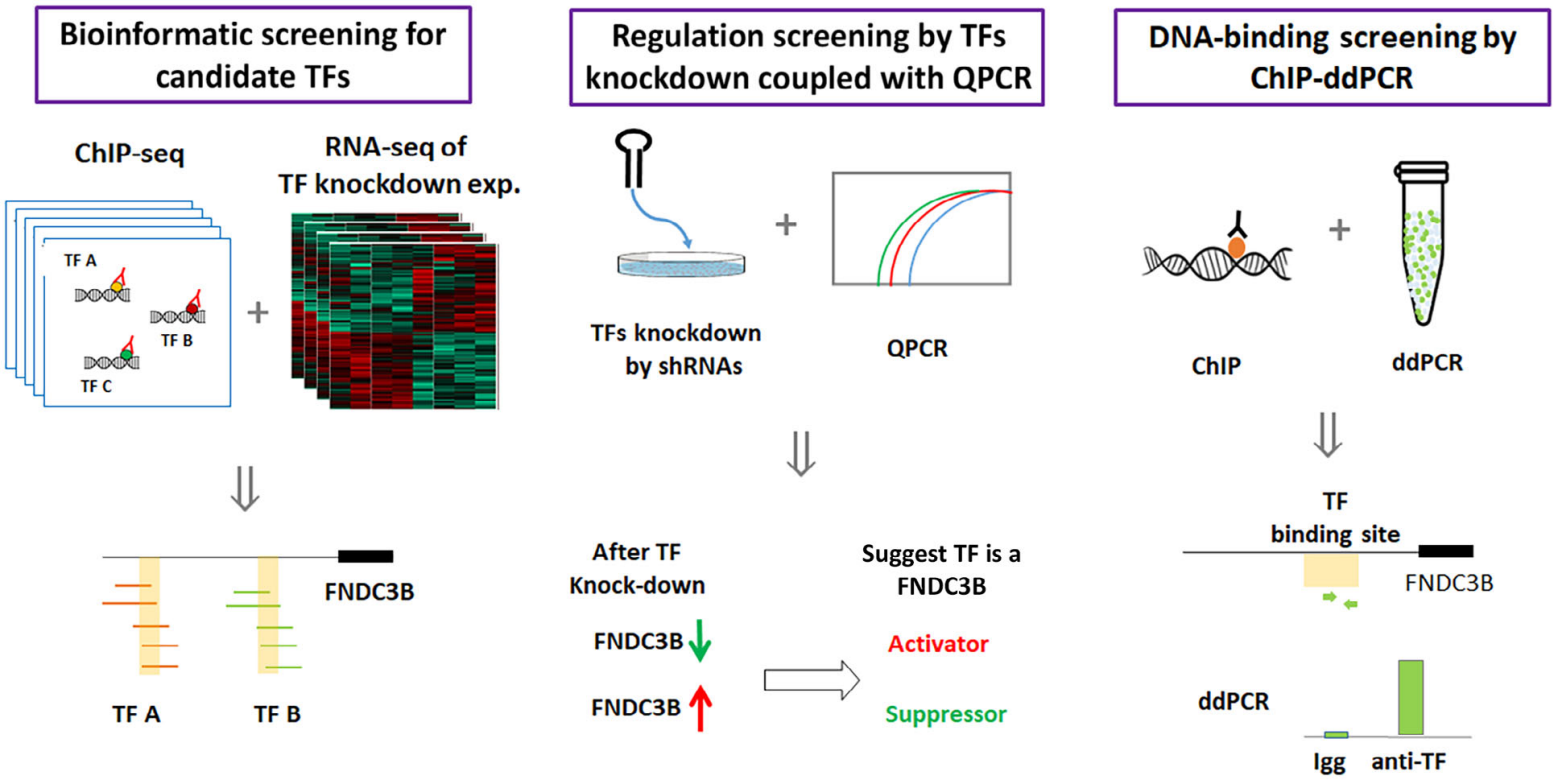
Flowchart of the integrated strategy used to reveal TFs for FNDC3B. There are three major steps for our TFs screening: bioinformation, regulation, and DNA‐binding. By summarizing the ChIP‐seq data from the public domain databases, candidate TFs were selected for further verification. TF knockdowns and qPCR were applied to validate the regulatory relationship between TFs and FNDC3B. ChIP–ddPCR was applied to verify that TFs could bind to the promoter of FNDC3B.

To export the TFs for FNDC3B, we established a series of bioinformatic, gene regulation, and DNA interaction approach to select our candidates (Fig. 1). Initially, we collected binding information of variant TFs from the ENCODE project. As candidate TFs, their binding regions should locate within 5 kb upstream of the transcription start site and support from more than one CHIP‐seq experiment was required. In total, we identified 53 TFs that meet our criteria. Since gene knockout technology has been widely applied recently, gene expression data from various TF knockout experiments can also be found and queried on the GEO database [27]. By reanalyzing the expression data from the GEO database, we eliminated 7 TFs from our list because their gene knockout did not result in any changes in FNDC3B expression.

### Regulation screening by TFs knockdown coupled with quantitative PCR

**Fig. 2:**
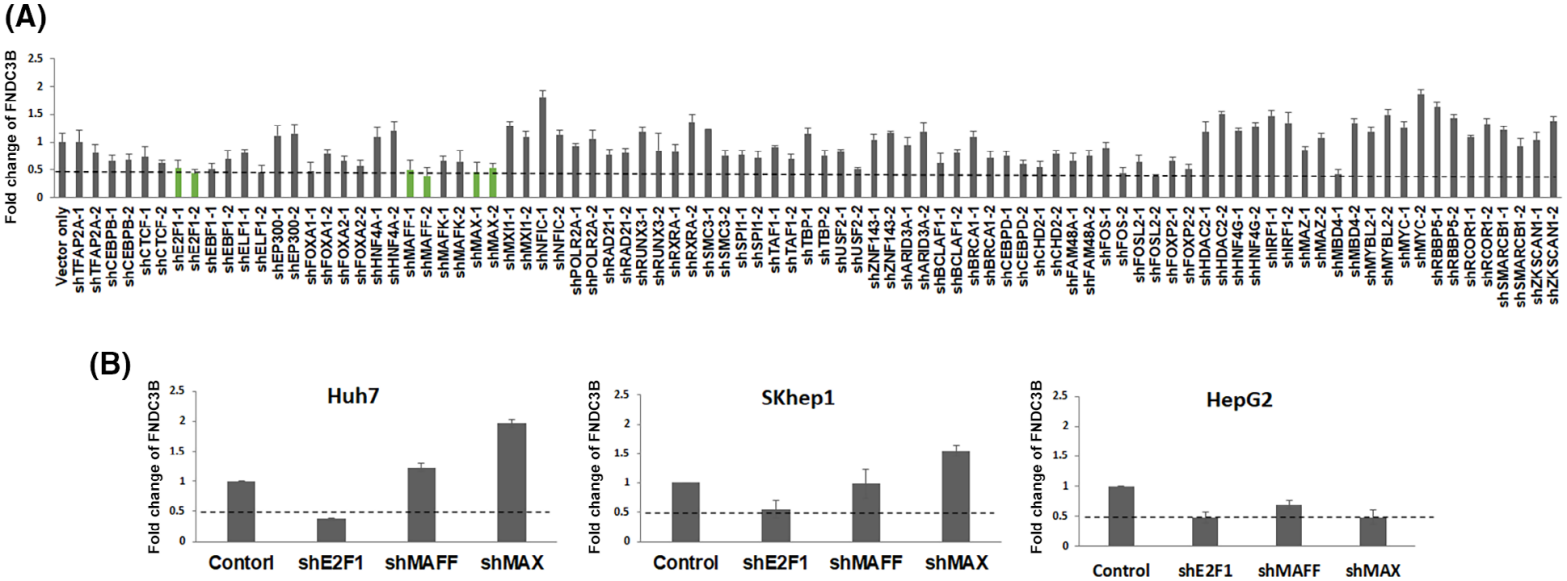
Regulation Screening by TFs Knockdown coupled with QPCR. (A) For each TF, two shRNAs were designed to transiently knock down its expression. qPCR was used to calculate the expression of FNDC3B, and the results are presented as relative fold changes. All the experiments were repeated three times independently and presented as mean ± SD. (B) The shRNA of E2F1, MAX, and MAFF were delivered by the lentivirus system in Huh7, SKhep1, and HrpG2 cells. The expression levels of FNDC3B were calculated using the $2^{-\Delta \Delta C_{q}}$ method, and the results are presented as relative fold changes. All the experiments were repeated three times independently and presented as mean ± SD.

To demonstrate the regulatory relationship between TF and FNDC3B, we designed two shRNAs for each TF to transiently knock down their expression. Subsequently, we measured the changes in FNDC3B expression levels using quantitative PCR (qPCR). If both shRNAs consistently increased or decreased the expression of FNDC3B, the TF was considered as regulator for FNDC3B. Our finding revealed that the knockdown of E2F1, MAFF, and MAX results in a reduction of FNDC3B in Huh7 cells (Fig. 2A). To further validate these findings, we employed a more efficient lentivirus system to deliver the shRNA. The results demonstrated that the expression of FNDC3B was consistently downregulated in three E2F1 knock down cells (Fig. 2B). Therefore, our results suggest that E2F1 was the transcriptional activator for FNDC3B.

### E2F1 binding sites prediction

**Fig. 3:**
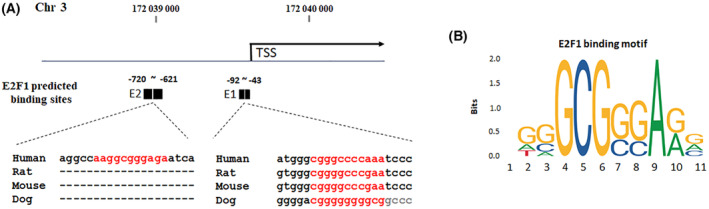
E2F1 predicted binding sites on the promoter of FNDC3B. (A) E2F1‐binding elements, E1 and E2, were predicted by GTRD (http://gtrd.biouml.org/). (B) JASPAR (http://jaspar.genereg.net/) E2F1 motif was used to determine the binding site in E1 and E2.

To refine the E2F1 binding regions on the promoter of FNDC3B, we utilized the TF binding information available from GTRD (http://gtrd.biouml.org/). GTRD integrates multiple ChIP‐seq experiment information and generates ChIP‐seq peaks using four different methods, which are then merged into clusters. Based on the GTRD predictions, we identified two E2F1 binding regions, namely E1 (−92 to −43) and E2 (−720 to −621), within the 5 kb upstream of transcription starting site (Fig. 3A). To assess the presence of E2F1 binding motifs in E1 and E2, we compared their sequences with known E2F1 binding motifs using JASPAR (http://jaspar.genereg.net/) (Fig. 3B). The results revealed the presence of E2F1 binding motifs in both E1 and E2. Notably, the sequence of E2F1 binding motif in E1 was highly conserved across multiple species. However, E2F1 binding motif sequence in E2 was only found in human.

### DNA‐binding screening by ChIP–droplet digital PCR (ddPCR)

**Fig. 4:**
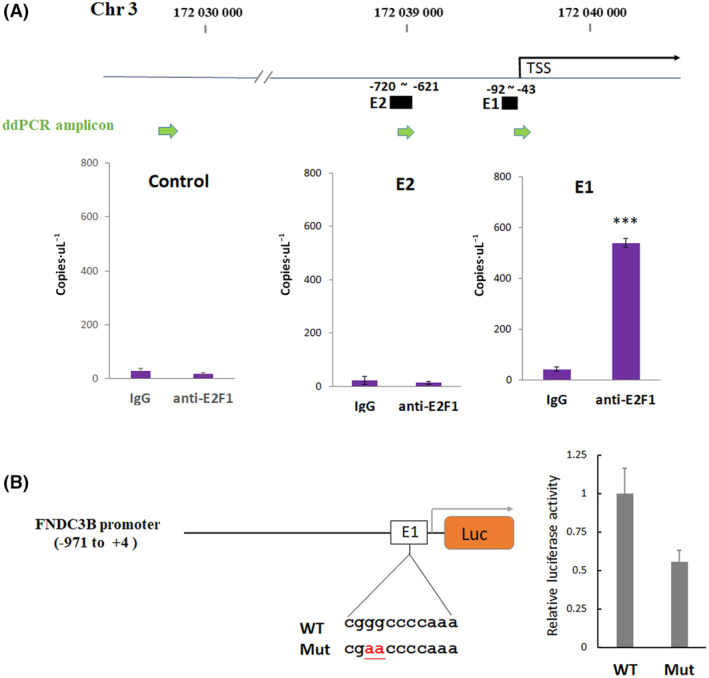
DNA‐binding screening. (A) PCR primer pairs were designed to amplify E1, E2, and a negative control fragment. The ddPCR was carried out with DNA fragments immunoprecipitated by anti‐E2F1 or IgG. Each PCR was repeated three times, and the error bars represent SD. *** represents an extremely significant difference (Student's *t*‐test, *P* < 0.001). (B) Dual‐luciferase reporter assay was used to detect E2F1 binding sites on the FNDC3B promoter region.

To validate the interaction between E2F1 and the FNDC3B promoter, we performed ChIP to isolate genomic DNA bound by E2F1. Traditionally, specific primer pairs are designed for qPCR to determine if the known binding sites are specifically enriched by immunoprecipitation. Our study employed a more advanced PCR system called ddPCR, recommended for accurately detecting the absolute number of DNA molecules present in a sample [28]. In our ChIP‐ddPCR experiments, we observed that E2F1 exhibited significant binding to E1 region compared to the E2 and non‐E2F1 binding control regions (Fig. 4A). To experimentally validate the role of E2F1 as a transcriptional activator for FNDC3B, we generated luciferase reporter constructs containing a segment of the FNDC3B promoter spanning from −971 to +4 bp. Within this construct, we specifically created mutation to disrupt the E2F1 binding site in E1. By comparing the luciferase activity, the mutated construct exhibited substantially diminished luciferase activity compared to wild‐type. These results indicated that E2F1 was directly bound with the FNDC3B promoter to activate the FNDC3B transcription (Fig. 4B).

### E2F1 regulates the expression and function of FNDC3B

**Fig. 5:**
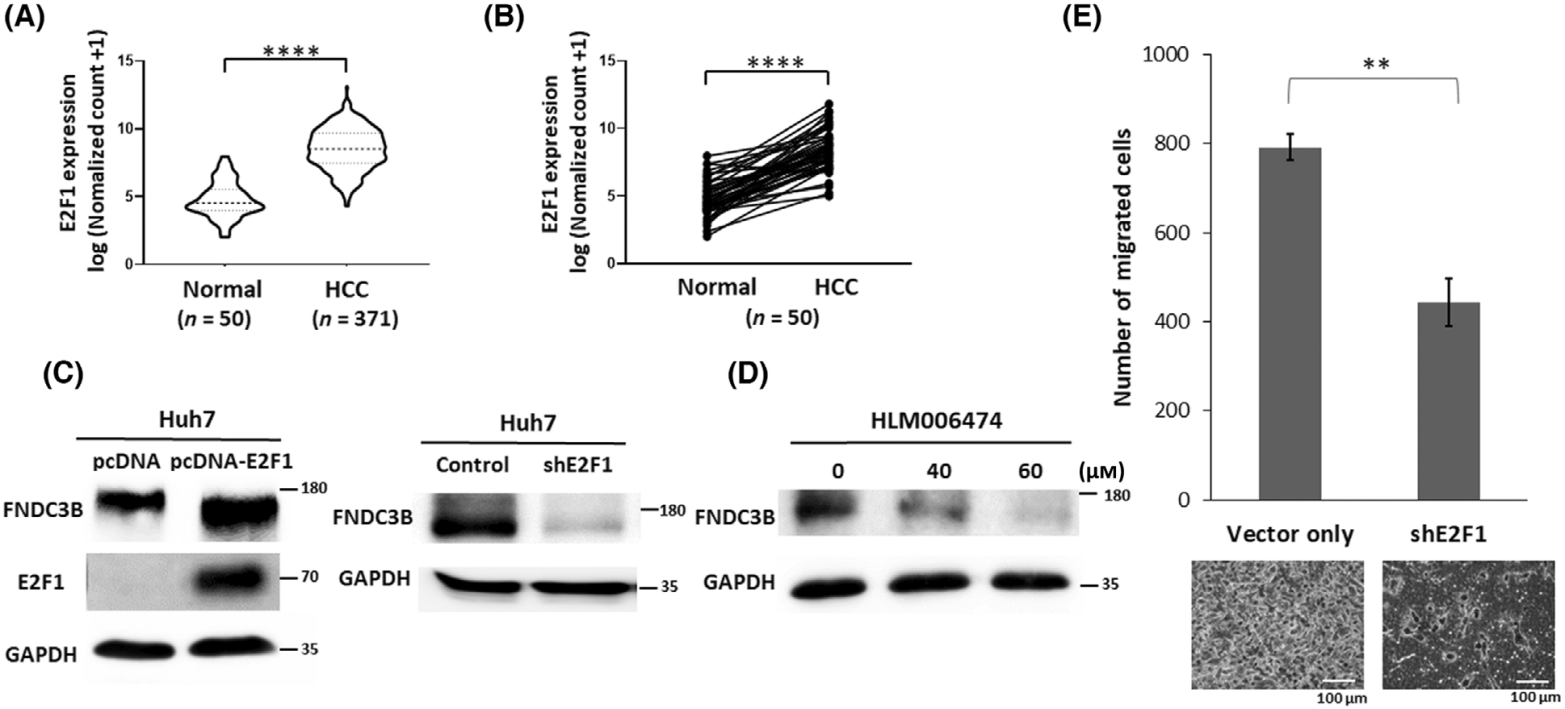
E2F1 regulated the expression and function of FNDC3B. (A) Violin plot showing the mRNA expression of E2F1 in HCC and normal tissues from the TCGA database. The dashed line and dotted lines within the violin plot represent the median and interquartile range, respectively. (B) E2F1 mRNA expression was upregulated in HCC tissues (**** for *P* < 0.0001) compared with 50 paired non‐cancerous adjacent tissues using Wilcoxon designed‐rank tests. (C) Western blot of FNDC3B in the E2F1 overexpressed and knockdown cells. Experiments were repeated three times. (D) Huh7 cells were treated for 24 h with the indicated concentration of HLM006474. Experiments were repeated three times. (E) Transwell migration assay performed using cells transfected with shE2F1. Migration ability was determined by calculating migration cells after 24 h of incubation. All the experiments were repeated three times independently and presented as mean ± SD. ** represent an extremely significant difference (Student's *t*‐test, *P* < 0.01). Scale bar: 100 μm.

Through the analysis of gene expression data from HCC patients in the TCGA database, we observed that E2F1 is highly expressed in tumors (Fig. 5A). Furthermore, analysis of E2F1 expression in 50 matched tumor and normal pairs demonstrated that E2F1 was significantly increased in tumor (Fig. 5B). Western blot analysis showed that overexpressed and knockdown of E2F1 significantly alter the expression of FNDC3B (Fig. 5C). E2F1 inhibitor (HLM006474) treatment also demonstrated that the E2F1 is required for the transcription of FNDC3B (Fig. 5D). Our previous studies have demonstrated that FNDC3B primarily plays a role in promoting cell migration during the tumorigenesis [9]. A transwell migration assay indicated that inhibition of E2F1 significantly suppresses cell migration ability (Fig. 5E). Overall, our findings suggested that the promotion of cell migration by FNDC3B is regulated by E2F1.

## Discussion

In this study, we employed a combination of bioinformatics, gene regulation, and DNA–protein interaction screening to investigate potential upstream TFs for FNDC3B. Firstly, we curated a list of candidate TFs based on previous ChIP‐seq experimental data. Subsequently, we performed shRNA knockdown experiments to identify TFs could impact FNDC3B expression. Our findings revealed a significant positive correlation between E2F1 and FNDC3B across various HCC cell lines. To further validate the regulatory relationship, we employed ChIP–ddPCR to validate E2F1 binding to the FNDC3B promoter. Additionally, through western blot and migration assays, we demonstrated that E2F1 can influence the expression and function of FNDC3B. This study established a high‐throughput and efficient method for transcription factor screening, contributing valuable insights into the future research on upstream regulatory factors for other genes.

TFs can be classified into two main categories: cis‐regulatory and trans‐regulatory factors. Cis‐regulatory factors are involved in regulating the transcription of nearby genes, whereas trans‐regulatory factors regulate the expression of distant genes [29, 30]. Both cis‐acting and trans‐acting factors play important roles in mediating gene expression. For the cis‐regulatory factors that commonly interact with promoters, it is easier to identify which TF controls the expression of nearby genes. In this study, our specific focus was on screening cis‐regulatory factors for FNDC3B. Finally, we successfully identify E2F1 as a transcriptional activator for FNDC3B. However, we do not exclude the possibility of other TFs serving as trans‐regulatory factors for FNDC3B.

E2F1, a member of the E2F family, is a transcription activator involved in various biological processes such as cell cycle regulation, cellular proliferation, and apoptosis [31, 32]. Numerous studies have demonstrated the carcinogenic role of E2F1 in HCC [33]. For example, E2F1 has been shown to induce HCC proliferation by activating PKCα phosphorylation [34]. Furthermore, E2F1 promotes HCC cell proliferation, migration, and invasion by activating DDX11 [35]. In our study, we made the novel discovery that FNDC3B transcription is activated by E2F1, and the knockdown of E2F1 leads to a decrease in FNDC3B levels in HCC cells. Additionally, our findings indicate that the effects of FNDC3B on cell migration are mediated by the activation of E2F1. These results contribute to a better understanding of the complex regulatory network involving E2F1 and FNDC3B in tumorigenesis of HCC.

In our results, the impact of overexpressed E2F1 on FNDC3B expression is less significant (Fig. 5C). Since the expression level of E2F1 in tumors is significantly higher than in normal tissues (Fig. 5A), we speculate that the expression level of E2F1 in HCC cells has already approached the maximally stimulated for FNDC3B production. Another possibility is that the expression of FNDC3B may be subject to feedback inhibition control. According to our observations, overexpression of FNDC3B in HCC cells can only be sustained for a short period. Eventually, endogenous FNDC3B is significantly inhibited, leading to total FNDC3B levels approaching the original baseline (data not shown). In this study, we did not identify any transcriptional inhibitors to support our hypothesis regarding feedback inhibition. However, several non‐coding RNAs have been found to regulate FNDC3B expression in other cancers recently [14, 36, 37]. We will further investigate the potential role of these non‐coding RNAs in feedback inhibition of FNDC3B expression.

In this study, our comprehensive approach, combining bioinformatics, regulation analysis, and DNA–protein interaction assays, led to the identification of E2F1 as a transcriptional activator of FNDC3B. Our findings provide valuable insights into the regulatory mechanisms and role of FNDC3B in tumorigenesis. This study contributes to a better understanding of the molecular pathways involved in cancer development and may have potential implications for the development of targeted therapies and interventions in the future.

## Conflict of interest

The authors declare no conflict of interest.

## Author contributions

KH, C‐TW, C‐HL performed the experiments. KH and C‐FC analyzed the data. C‐FC designed and supervised the study. KH and C‐FC wrote the manuscript.

## Data Availability

The datasets generated and/or analyzed during the current study are available from the corresponding author (cfchen@nycu.edu.tw) upon reasonable request.
